# Impaired B-Cell Differentiation in a Patient With *STAT1* Gain-of-Function Mutation

**DOI:** 10.3389/fimmu.2020.557521

**Published:** 2020-09-29

**Authors:** Kazuki Nemoto, Toshinori Kawanami, Takayuki Hoshina, Masataka Ishimura, Kei Yamasaki, Satoshi Okada, Hirokazu Kanegane, Kazuhiro Yatera, Koichi Kusuhara

**Affiliations:** ^1^ Department of Respiratory Medicine, School of Medicine, University of Occupational and Environmental Health, Japan, Kitakyushu, Japan; ^2^ Department of Pediatrics, School of Medicine, University of Occupational and Environmental Health, Japan, Kitakyushu, Japan; ^3^ Department of Pediatrics, Graduate School of Medical Sciences, Kyushu University, Fukuoka, Japan; ^4^ Department of Pediatrics, Hiroshima University Graduate School of Biomedical and Health Sciences, Hiroshima, Japan; ^5^ Department of Child Health and Development, Graduate School of Medical and Dental Sciences, Tokyo Medical and Dental University, Tokyo, Japan

**Keywords:** *STAT1* gain-of-function mutation, disseminated cryptococcosis, hypogammaglobulinemia, impaired B-cell differentiation, bone marrow (BM)

## Abstract

Hypogammaglobulinemia is a rare complication of *STAT1* gain-of-function (GOF) mutations. We report an adult patient diagnosed with hypogammaglobulinemia caused by B-cell depletion during the treatment of disseminated cryptococcosis. The patient carried the *STAT1* GOF mutation (c.820C>T, p.R274W). The flow cytometric analysis of his bone marrow revealed that B-cell differentiation was blocked in the stages between pre-B1b and pre-B2 cells. On the other hand, his brother who carried the same mutation displayed normal B-cell counts, thereby indicating that the unrecognized variants in same or other gene might be associated with abnormal B-cell differentiation in the patients. In conclusion, impaired B-cell differentiation in the bone marrow can cause hypogammaglobulinemia in patients with *STAT1* GOF mutations.

## Introduction

Gain-of-function (GOF) mutations in the signal transducer and activator of transcription 1 (*STAT1*) gene are recognized as genetic abnormalities that cause chronic mucocutaneous candidiasis disease (CMCD) (1, 2). This autosomal dominant immunodeficiency is characterized by recurrent or persistent mucocutaneous lesions, such as on the nails, skin, and oral or genital mucosae, caused by the *Candida* species, and some patients with this disorder develop invasive fungal infections (3). Low B-cell count (19%) and low immunoglobulin (Ig) G levels (3%) have been observed as rare immunological abnormalities in patients with *STAT1* GOF mutations (3). Although the mechanisms underlying B-cell lymphopenia and hypogammaglobulinemia have previously been investigated (4), they are still somewhat elusive.

We herein report an adult patient diagnosed with *STAT 1* GOF mutation-induced hypogammaglobulinemia caused by B-cell depletion during the treatment of disseminated cryptococcosis. To the best of our knowledge, this is the first cases report that demonstrated the impairment of B-cell differentiation in bone marrow of such a patient.

## Case Presentation

A 30-year-old Japanese man was admitted to our hospital because of high-grade fever with transient consciousness disorder and double vision. He had no medical history throughout his childhood and adolescence but continued to have repeated fever and productive cough since the age of 25. He also presented with stomatitis and dental caries. His mother had a history of disseminated cryptococcosis and died from chronic hepatitis C virus infection. His younger brother had been clinically diagnosed with CMCD for having recurrent and refractory cutaneous candidiasis although no genetic analysis has been performed on his dissent.

**Figure 1:**
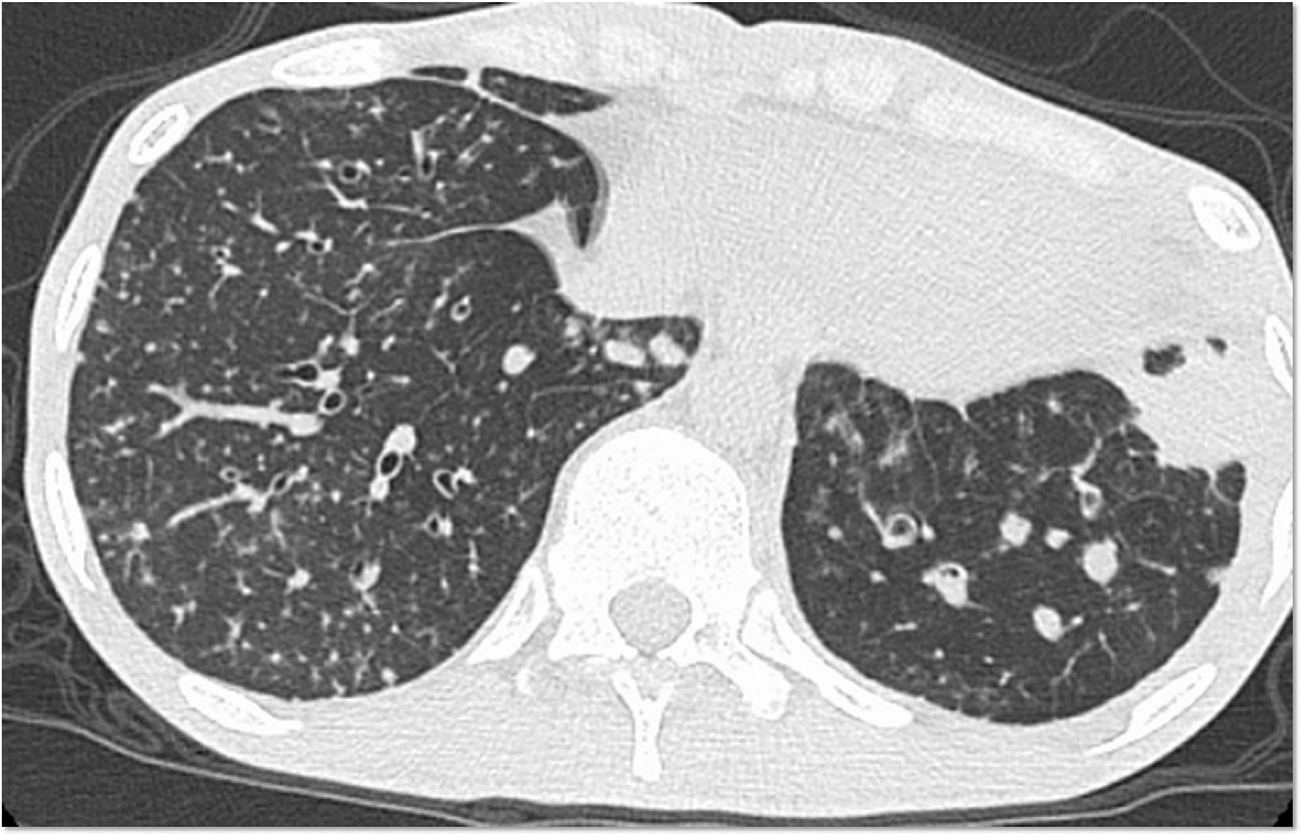
Chest computed tomography scan. Bronchiectasis and multiple granular shadows are randomly distributed in the bilateral lobes, and consolidation in the lower left lobe is present.

Physical examination on the admission revealed that the majority of his teeth were corroded, and that he had white moss throughout his mouth. A nuchal stiffness was noted with right abductor paralysis. His chest computed tomography (CT) showed bronchiectasis and multiple granular shadows randomly distributed in the bilateral lower and middle lobes, thin-walled cysts predominantly in both upper lobes, and consolidation in the lower left lobe (**Figure 1**). *Cryptococcus neoformans* was isolated from his blood and cerebrospinal fluid samples and was also detected in liver and lung biopsies. Accordingly, he was diagnosed with disseminated cryptococcosis with meningitis and oral and cutaneous candidiasis.

The patient was treated with a combination therapy of liposomal amphotericin B and flucytosine for 8 weeks. Consequently, his respiratory symptoms and neurological abnormalities resolved. The cryptococcal antigen titers in his cerebrospinal fluid decreased from 1,024 times to 32. His chest CT on day 44 of the treatment showed that the granular shadows and consolidation entirely improved, but the bronchiectases in his right middle lobe and left lingular segment remained. The antifungal treatment was then switched to oral fluconazole, and the disease did not recur for one year.

**Figure 2:**
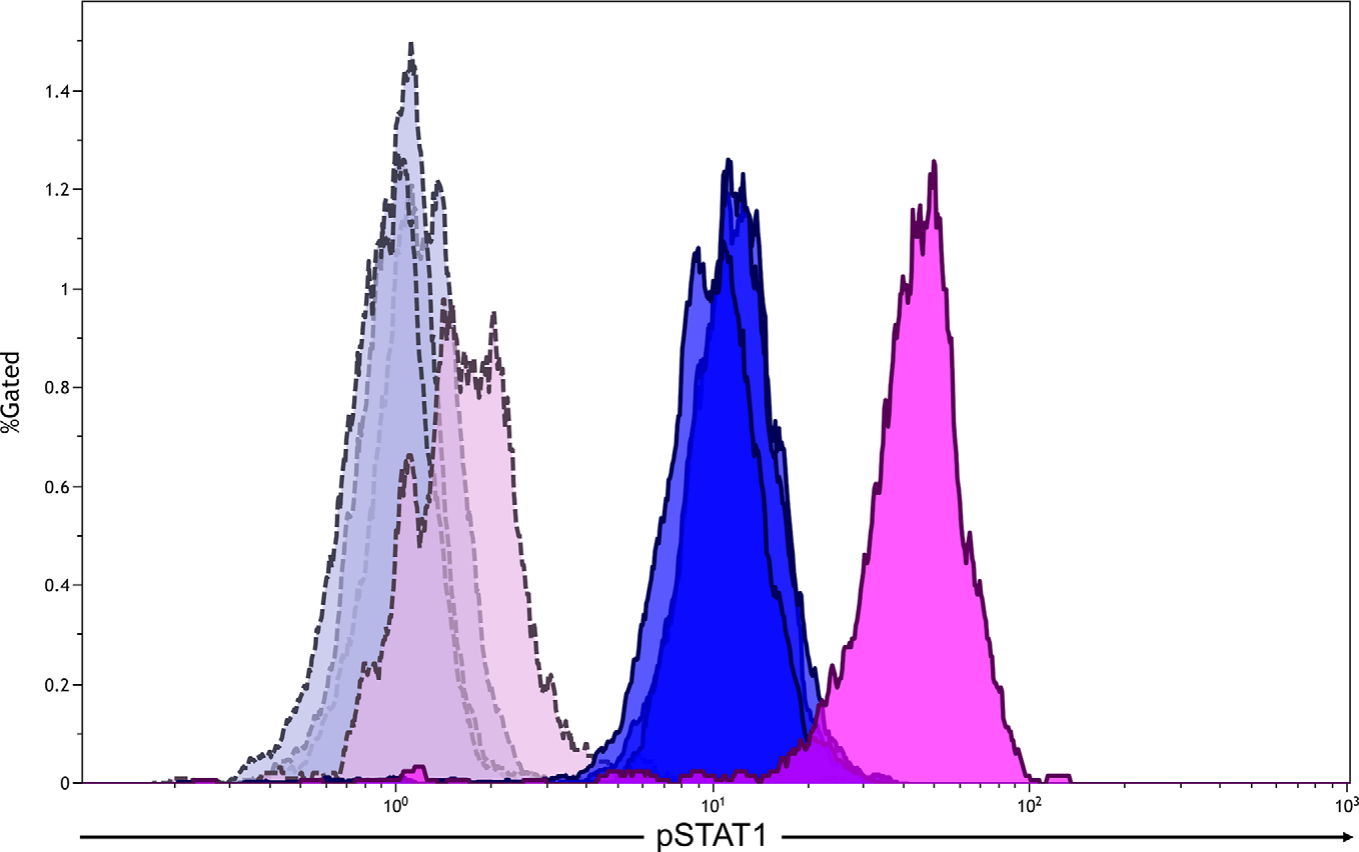
Flow cytometric analysis of phosphorylationed STAT1 (pSTAT1) in monocytes after the stimulation with interferon-*γ* (500 U/mL). The analysis gate was set in CD14^+^ cells. Blue areas indicate healthy adults (n = 3), whereas pink areas indicate the patient. Gray and purple areas indicate pSTAT1 in monocytes without interferon-*γ* stimulation in healthy adults and the patient, respectively.

The patient was suspected of having primary immunodeficiency because of the present clinical course and his family history. We sequenced the patient’s CMCD-related genes and found a previously recognized heterozygous missense mutation in the coiled-coil domain of the *STAT1* gene (c.820C>T, p.R274W) (5, 6). The same mutation was also detected in his younger brother. The monocytes of the patient showed hyper-phosphorylation of STAT1 after stimulation with interferon-*γ* (**Figure 2**). Based on these results, the patient was diagnosed with STAT1 GOF.

**Figure 3:**
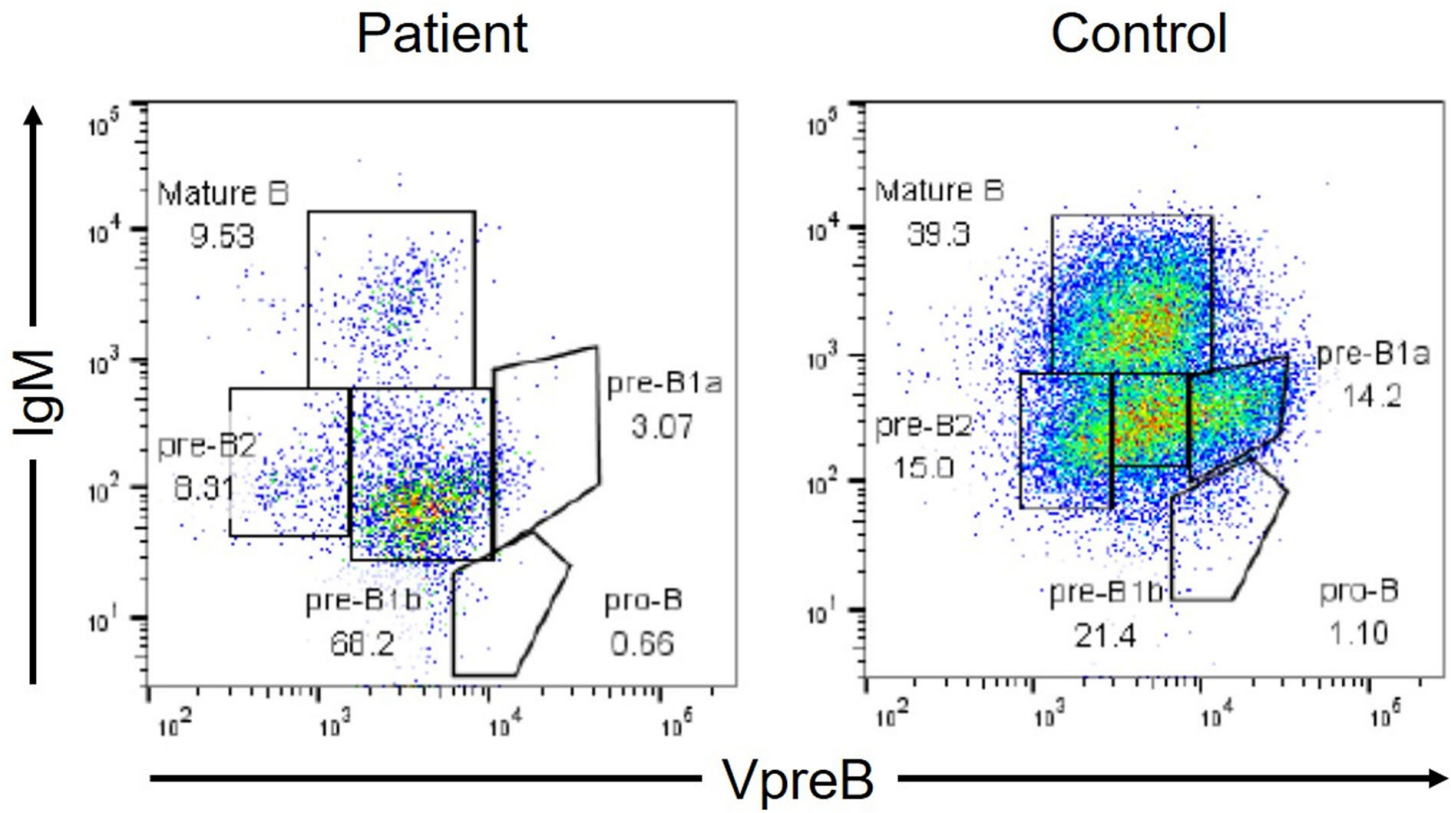
Flow cytometric analysis of B-cell differentiation in the bone marrow (BM). VpreB is a component of the surrogate light chains. The analysis gate was set in CD19^+^ and/or VpreB^+^ cells. The BM cells were fixed and permeabilized to simultaneously analyze the cell surface antigens (IgM and CD19) and the intracellular molecule (VpreB).

Additionally, the patient presented with low serum IgG and IgA levels [IgG: 2.36 g/L, normal range (NR): 8.61–17.47 g/L; IgA: 0.05 g/L, NR: 0.93–3.93; IgM: 0.92 g/L, NR: 0.33–1.83]. Surface marker analyses revealed that 72.3% (NR: 54.3–81.9%) of his peripheral lymphocytes comprised CD3^+^ cells with a CD4/CD8 ratio of 32.8 (NR: 24.3–49.7%)/36.2 (NR: 18.4–49.0%), and that 0.1% (NR: 2.9–20.1%) comprised CD19^+^ cells (**Table 1**). Peripheral blood lymphocyte responsiveness to phytohemagglutinin was normal (Stimulation Index: 281, NR: 254–388). The level of T-cell receptor excision circles was 2.06 x 10^2^ copies/μgDNA [NR: 3.4 ± 3.6 x 10^2^ copies/μg DNA (7)], whereas both those of the coding joint and signal joint kappa-deleting recombination excision circles were undetermined. We then investigated the cause of hypogammaglobulinemia and B-cell lymphopenia. The B-cell differentiation was blocked in the stages between VpreB^+^IgM^low^ (pre-B1b) cells and VpreB^−^IgM^low^ (pre-B2) cells in the bone marrow sample obtained three months after the onset of cryptococcosis (**Figure 3**). Human immunodeficiency virus-RNA and Epstein-Barr virus- and cytomegalovirus-DNA were undetectable by polymerase chain reaction. No pathogenic variants in antibody deficiency-causing genes listed in the 2017 Primary Immunodeficiency Diseases Committee Report on Inborn Errors of Immunity were detected using the target panel sequence (8). The reduction in the peripheral B-cell counts had persisted even after the patient’s recovery from cryptococcosis (**Table 1**). His prolonged respiratory symptoms did not recur upon regular subcutaneous immunoglobulin infusion to maintain a trough level of >7.0 g/L.

**Table 1:** The analysis of lymphocyte subsets in the acute phase of cryptococcosis and the non-infectious state.

	Acute phase of the infection	Non-infectious state^*1^	Normal range
White blood cell counts, x 10^3^/μL	2.80	4.00	3.30–8.60
Lymphocyte counts, x 10^3^/μL	0.69	1.60	1.06–3.01
CD3^+^ cells, % in lymphocytes	72.3	77.7	54.3–81.9
CD19^+^ cells, % in lymphocytes	0.10	0.22	2.9–20.1

^*1^The sample was obtained 1.5 year after the onset of cryptococcosis.

## Discussion

Disseminated cryptococcosis is a fungal infection that frequently develops in patients with *STAT1* GOF mutations (3). Th17 cells are thought to play an important role in the protection against cryptococcal infections (9, 10). The STAT1 signaling is overactive in patients with *STAT1* GOF mutations. Increased STAT1 function inversely leads to the impaired production of STAT3-dependent cytokines, including interleukin (IL)-17A and IL-17F, in T cells. The present patients developed disseminated cryptococcosis as the initial manifestation of *STAT1* GOF mutation, suggesting that this mutation should be considered for differential diagnosis, even if a previously-healthy adult develops disseminated cryptococcosis, because this genetic disorder may, although rarely, develop in adulthood (3, 5).

The novelty of this case report lies in the analysis of B-cell differentiation of the bone marrow sample of a patient with *STAT1* GOF mutation. The mechanistic details of such impaired Th17 responses are accordingly somewhat understood, whereas those of impaired humoral immunity caused by B-cell lymphopenia in patients with *STAT1* GOF mutations are still elusive because of the small number of patients with humoral immunodeficiency (3, 5). A previous study analyzed the peripheral mature B-cells and consequently suggested that the accelerated B-cell apoptosis resulting from increased caspase activity might cause B-cell lymphopenia (4). However, no study has investigated B-cell differentiation in the bone marrow of patients with the *STAT1* GOF mutations accompanying B-cell lymphopenia. It has been speculated that *Btk* mutations in X-linked agammaglobulinemia (XLA) may interrupt the proliferation and survival of the earliest pre-B cells (pre-B1a cells) (11). STAT3 whose functions are inhibited in patients with the *STAT1* GOF mutations is also associated with early B-cell development (earlier stage of pro-B cells) in the bone marrow (12). We observed that B-cell differentiation in such a patient was blocked in the stages between the pre-B1b and pre-B2 cells of the bone marrow, which was later than in patients with XLA. Furthermore, the STAT1-dependent B-cell differentiation is affected by infection. In mice, STAT1 plays an important role in the promotion, but not the impairment, of B-cell differentiation in the bone marrow and marginal zone (13, 14). In our patient, the reduction in the peripheral B-cell counts persisted even after his recovery from cryptococcosis, thereby indicating that the impaired B-cell differentiation was not caused by infection. Although the molecular biological analysis to elucidate the mechanism of the impaired B-cell differentiation was performed, we believe that B-cell differentiation in patients with *STAT1* GOF mutations is interrupted by a pathway other than the BTK- and STAT3-associated pathways.

The decreased number of B cells is only observed in some the patients with such mutations. In fact, the B-cell counts and the immunoglobulin levels of the patient’s younger brother were within normal ranges (data not shown). Although no pathogenic variants in previously recognized antibody deficiency-causing genes (8) have been detected in our patient, unrecognized variants in same or other gene may have caused the impaired B-cell differentiation in the bone marrow. Indeed, a recent study has suggested that the interplay between rare and common genetic variants may provide the phenotypic heterogeneity of primary immunodeficiency (15). Whole-genome sequencing is a future prospective to elucidate genetic difference between the siblings, thus providing a better understanding of the molecular mechanisms underlying impaired B-cell differentiation. Alternatively, the reduction in the B-cell counts observed in patients with the *STAT1* GOF mutations may depend on age. Our patient might have developed hypogammaglobulinemia in adulthood because his respiratory symptoms appeared 5 years before the present episode. Further studies are needed to elucidate the details of the mechanism underlying the impaired B-cell differentiation in this disorder.

## Data Availability Statement

The datasets presented in this study can be found in online repositories. The names of the repository/repositories and accession number(s) can be found in the article/supplementary material.

## Ethics Statement

Written informed consent was obtained from the patient for the publication of any potentially identifiable images or data included in this article.

## Author Contributions

KN, TK, and TH were involved in the treatment of the patient, conceptualized the study, carried out the initial analysis of data for work, and drafted the initial manuscript. KeY and KaY were involved in the treatment of the patient. MI, HK, and SO carried out the acquisition and analysis of data for the work. KK coordinated and supervised data collection. All authors contributed to the article and approved the submitted version.

## Conflict of Interest

The authors declare that the research was conducted in the absence of any commercial or financial relationships that could be construed as a potential conflict of interest.
